# Shannon Entropy Computations in Navier–Stokes Flow Problems Using the Stochastic Finite Volume Method

**DOI:** 10.3390/e27010067

**Published:** 2025-01-14

**Authors:** Marcin Kamiński, Rafał Leszek Ossowski

**Affiliations:** Faculty of Civil Engineering, Architecture and Environmental Engineering, Lodz University of Technology, 90-924 Łódź, Poland; rafal.ossowski@gmail.com

**Keywords:** stochastic finite volume method, Shannon entropy, Navier–Stokes equations, stochastic perturbation technique.cx

## Abstract

The main aim of this study is to achieve the numerical solution for the Navier–Stokes equations for incompressible, non-turbulent, and subsonic fluid flows with some Gaussian physical uncertainties. The higher-order stochastic finite volume method (SFVM), implemented according to the iterative generalized stochastic perturbation technique and the Monte Carlo scheme, are engaged for this purpose. It is implemented with the aid of the polynomial bases for the pressure–velocity–temperature (PVT) solutions, for which the weighted least squares method (WLSM) algorithm is applicable. The deterministic problem is solved using the freeware OpenFVM, the computer algebra software MAPLE 2019 is employed for the LSM local fittings, and the resulting probabilistic quantities are computed. The first two probabilistic moments, as well as the Shannon entropy spatial distributions, are determined with this apparatus and visualized in the FEPlot software. This approach is validated using the 2D heat conduction benchmark test and then applied for the probabilistic version of the 3D coupled lid-driven cavity flow analysis. Such an implementation of the SFVM is applied to model the 2D lid-driven cavity flow problem for statistically homogeneous fluid with limited uncertainty in its viscosity and heat conductivity. Further numerical extension of this technique is seen in an application of the artificial neural networks, where polynomial approximation may be replaced automatically by some optimal, and not necessarily polynomial, bases.

## 1. Introduction

Uncertainty in fluid flows is remarkably less frequently studied in computational mechanics [1] than in solids and structure deformations [2,3], but following physical observations, it seems to be more natural and accurate. Further, it is usually characterized by decisively larger statistical scattering of state-dependent physical parameters, so that uncertainty quantification and/or propagation may exhibit unpredictable and highly nonlinear characteristics. Uncertainty quantification in both fluids and solids is traditionally delivered with the help of various implementations of the stochastic finite element method (SFEM) [4], whereas analogous extensions of other discrete numerical methods are definitely less popular, and even scarce. This specifically applies to the finite volume method (FVM) [5,6], which seems to be more efficient than the FEM in many fluid flow problems described by the Navier–Stokes equations [7,8,9,10]. The FVM is decisively less popular in computational mechanics than the FEM, but its implementations and applications in stress analysis [11], multiscale analyses [12,13], and heterogeneous media [14] make this methodology more attractive, useful, and accurate. Common usage of the FVM with machine learning algorithms [15], along with new research paths including adaptivity [16] or mimetic algorithms [17], are observed. There is well documented evidence in the literature of the application of this method to solve a huge variety of stochastic problems, e.g., conservation laws in physical systems [18], computer science issues [19], flow problems [10], and energy simulations [20].

It is well known that modern stochastic analysis is usually based upon determination of the basic probabilistic moments and coefficients of the state functions for the given computational domain [21], including recently developed methods with fractional derivatives [22]. This enables the further discussion and justification of statistical correlations between different mechanical and physical fields in regards to reliability analysis [23]. Direct determination of the resulting probability functions in the given system, exhibiting some random parameters, is still explored, i.e., Ref. [24]; nevertheless an alternative method consists of the estimation (or direct computation) of probabilistic entropy. It can be determined using discrete or continuous probability measures for the physical quantity of interest. Shannon entropy [25,26,27] is most frequently applicable to assess complexity, diversity, disorder, or chaos, and some mathematical models applied successfully in image analysis [28,29], computer science [30], information theory [26,31], economics [32], physics, and engineering [33,34], and employing this entropy may attract increasing interest and importance.

Therefore, the main idea of this work is a utilization of the stochastic finite volume method (SFVM) [35] for the determination of the spatial distribution of the Shannon theory for fully coupled Navier–Stokes equations relevant to the fluid flow problems in the presence of specific physical uncertainty. This is not the first attempt to include entropy application in fluid flows analysis [34,36]; however, the approach proposed combines different computational strategies to achieve this goal and to deliver a comparison of the entropy-based method with the existing moments-based approaches. It is based upon combined polynomial interpolation and the weighted least squares method (WLSM) recovery of polynomial bases, linking the PVT solutions with the physical parameters of the analyzed fluid. Random polynomials of some physical characteristics of the fluid are used in the stochastic perturbation method for Taylor expansions to calculate probabilistic moments in the given flow problem [4]. These polynomials are next engaged in Monte Carlo simulations (MCS), enabling Shannon entropy computations at the discrete finite volume level. Two numerical examples are presented here, namely (i) heat transfer in some trapezoidal plates and (ii) the 3D lid-driven cavity flow benchmark CFD, where Gaussian heat conductivity is considered. These two case studies enable a contrast of the first two probabilistic moments maps with those representing Shannon entropy, as well as analysis of the probabilistic convergence of all probabilistic responses, while increasing the size of the generated population in the MCS approach. These studies confirm several observations made in computational solid mechanics analyses, especially the fact that extreme values of the resulting coefficients of variation of the PVT solution coincide very well with those of Shannon entropy. Additionally, this work documents a coincidence of the maps for these two parameters, which may affect further studies in computational mechanics.

## 2. Uncertainty in Navier–Stokes Equations

The system of basic equilibrium Navier–Stokes equations, to be extended towards stochastic analysis and to be solved numerically, can be written in the given computational domain Ω for the unknown fluid state functions, i.e., velocities, *v_i_* = *v_i_(**x**)*; pressure, *p = p(**x**);* temperature, *θ = θ(**x**)* (x∈Ω), as follows [37]:

(1) $$\rho \left(\frac{\partial v_{i}}{\partial t}+v_{i,j}v_{j}\right)=\sigma_{ij,j}+{\tilde{f}}_{i},$$

(2) $$v_{i,i}=0,$$

(3) $$\sigma_{ij}=-p\delta_{ij}+2\mu \varepsilon_{ij},$$

(4) $$\rho c\left(\frac{\partial \theta }{\partial t}+\theta_{,i}v_{i}\right)={\left(k\theta_{,i}\right)}_{,i}+{\tilde{q}}_{i},$$

where the following geometrical equation is adopted:

(5) $$\varepsilon_{ij}=\frac{1}{2}\left(v_{i,j}+v_{j,i}\right)=\frac{1}{2}\;\left(\frac{\partial v_{i}}{\partial x_{j}}+\frac{\partial v_{j}}{\partial x_{i}}\right),\;\;\;i=1,2,3.$$

Here, the second-order symmetric tensors $\sigma_{ij}$, $\varepsilon_{ij}$ represent the stress and strain states for the given fluid, whose viscosity is traditionally denoted by *μ*, its heat conductivity by *k*, its heat capacity as *c*, and the mass density as *ρ*. These equations are relevant to the macroscopically homogeneous single-phase fluid, although they may also be rewritten and solved in case of any heterogeneity. The following general boundary conditions need to be imposed here:

(6) $$v_{i}={\hat{v}}_{i};x\in \partial \Omega_{v},$$

(7) $$\sigma_{ij}n_{j}={\hat{f}}_{i};x\in \partial \Omega_{\sigma },$$

(8) $$\theta =\hat{\theta };x\in \partial \Omega_{\Theta }$$

(9) $$k\frac{\partial \theta }{\partial x}=\hat{q};x\in \partial \Omega_{q}.$$

Variational formulation of this problem is introduced in the following way:

(10) $$\int_{\Omega }\delta v_{i}\rho \left({\dot{v}}_{i}+v_{i,j}v_{j}\right)d\Omega +\int_{\Omega }\delta v_{i,j}\left(2\mu \varepsilon_{ij}-p\delta_{ij}\right)d\Omega =\int_{\Omega }\delta v_{i}{\tilde{f}}_{i}d\Omega +\int_{\partial \Omega }\delta v_{i}{\hat{f}}_{i}d\left(\partial \Omega_{\sigma }\right).$$

(11) $$\int_{\Omega }\delta pv_{i,i}d\Omega =0,$$

(12) $$\int_{\Omega }\delta \theta \rho c\left(\dot{\theta }+\theta_{,i}v_{i}\right)d\Omega +\int_{\Omega }k\delta \theta_{,i}\theta_{,i}d\Omega =\int_{\Omega }\delta \theta \tilde{q}d\Omega +\int_{\partial \Omega_{q}}\delta \hat{\theta }\hat{q}d\left(\partial \Omega \right).$$

It is further assumed that some physical parameters of the analyzed fluid exhibit Gaussian uncertainty within the first two given moments [38]. An extension of the aforementioned equations to uncertainty analysis involves using the generalized stochastic perturbation technique. For this purpose, let us consider a random variable *b* and its probability density function (PDF) by *p_b_*(*x*) so that its expectation can be defined as follows:

(13) $$E\left(b\right)=\int_{-\infty }^{+\infty }x\;p_{b}\left(x\right)\;dx$$

assuming no additional truncation, in this case. Further, one can define the central probabilistic moment of the *m*th order as follows:

(14) $$\mu_{m}\left(b\right)=\int_{-\infty }^{+\infty }{\left(b-E\left[b\right]\right)}^{m}p_{b}\left(x\right)dx.$$

Let us consider the following representation of the random function **v**(*b*) with respect to its parameter *b* around its mean value [4,35], as follows:

(15) $$v\left(b\right)=v^{0}\left(b^{0}\right)+\varepsilon {\left.\frac{\partial v\left(b\right)}{\partial b}\right|}_{b=b^{0}}\Delta b+\ldots +\frac{\varepsilon^{n}}{n!}{\left.\frac{\partial^{n}v\left(b\right)}{\partial b^{n}}\right|}_{b=b^{0}}\Delta b^{n}$$

where ε is a given perturbation parameter, while the *n*th-order variation of a random variable is given as follows:

(16) $$\varepsilon^{n}\Delta b^{n}={\left(\delta b\right)}^{n}=\varepsilon^{n}{\left(b-b^{0}\right)}^{n}$$

Taking into account the partial derivatives of physical system function **v**(*b*), this function should be continuous and differentiable at the mean value of the input random parameter *b* = *b*^0^, which condition is fulfilled using polynomial, power, hyperbolic, and harmonic, as well as exponential, functions. Then, the expected values are evaluated from Equation (13) using the 10th-order expansion, with *ε* = 1, as follows:

(17) $$\begin{array}{l} E\left[v\left(b\right)\right]=v^{0}\left(b^{0}\right)+\frac{1}{2}{\left.\frac{\partial^{2}v\left(b\right)}{\partial b^{2}}\right|}_{b=b^{0}}\mu_{2}\left(b^{0}\right)+\frac{1}{4!}{\left.\frac{\partial^{4}v\left(b\right)}{\partial b^{4}}\right|}_{b=b^{0}}\mu_{4}\left(b^{0}\right)\\ +\frac{1}{6!}{\left.\frac{\partial^{6}v\left(b\right)}{\partial b^{6}}\right|}_{b=b^{0}}\mu_{6}\left(b^{0}\right)+\frac{1}{8!}{\left.\frac{\partial^{8}v\left(b\right)}{\partial b^{8}}\right|}_{b=b^{0}}\mu_{8}\left(b^{0}\right)+\frac{1}{10!}{\left.\frac{\partial^{10}v\left(b\right)}{\partial b^{10}}\right|}_{b=b^{0}}\mu_{10}\left(b^{0}\right) \end{array}$$

It is assumed that the input random variable *b* exhibits symmetrical, but not necessarily Gaussian, distribution to eliminate all odd-order term expansion components (uniform or symmetric triangular distributions are admissible here as well).

A precision of the expected value determination (and also variances) using this tenth-order approach has been demonstrated in the first numerical experiment. Assuming that the variable *b* exhibits Gaussian distribution, one may simply derive its higher-order central probabilistic moments in the following way:

(18) $$\mu_{p}\left(b\right)=\left\{\begin{array}{c} \;\;\;\;\;\;\;\;\;0;\;\;\;\;\;\;\;\;\;p=2k+1\\ {\left\{\sigma \left(b\right)\right\}}^{p}\left(p-1\right)!!={\left\{\sigma \left(b\right)\right\}}^{p}\left(p-1\right)\cdot \left(p-3\right)\cdot \ldots .\cdot 5\cdot 3;\;p=2k \end{array}\right.$$

for any natural k≥1, the expansions relevant to higher-order statistics in this methodology can be found in Ref. [4]. Uncertainty analysis, based upon probabilistic moments and coefficients, has some well-known limitations and may be biased due to some unpredictable numerical errors; therefore, a concept of probabilistic entropy has been proposed by Shannon [25] and then extended by many researchers. It states that uncertainty in the given technical system, quantified by a real valued function *f* = *f*(*b*), where *b* is this uncertainty source, may be quantified by a real number *h*, as follows:

(19) $$h\left(f\left(b\right)\right)=-\sum_{i=1}^{n}p_{i}\left(f\left(b\right)\right)\ln\left(p_{i}\left(f\left(b\right)\right)\right)\;$$

where *n* stands here for the number of possible different states of this system. Because a coefficient of variation (CoV) was dominantly used in stochastic computational mechanics to discuss uncertainty importance and propagation in the given boundary value problem, a comparison of the Shannon entropy distribution with the analogous distribution of the CoV for the given benchmark problem is delivered in the following sections.

## 3. Stochastic Finite Volume Method

The following polynomial basis is proposed for the resulting discrete temperature field $T_{\beta }$ in the presence of an uncertainty source *b* [4]:

(20) $$T_{\beta }\;=\;D_{\beta m}^{T}b^{m},\;\;m=0,\ldots ,n-1;\beta =1,\ldots ,N,$$

where $D_{\beta m}^{T}$ stands for a rectangular matrix of the unknown polynomial coefficients, so that the following continuous approximation is adopted:

(21) $$\theta \left(x_{i}\right)=\;\phi_{\beta }\left(x_{i}\right)\;\;T_{\beta }=\phi_{\beta }\left(x_{i}\right)\;\;D_{\beta m}^{T}b^{m},\;\;\;i=1,\;2;\beta =1,\;2,\ldots ,N;m=0,\ldots ,n-1;$$

where $\phi_{\beta }$ and $T_{\beta }$ are traditional deterministic shape functions. The temperature gradients are similarly determined as follows:

(22) $$\theta_{,j}=\phi_{\beta ,j}\;T_{\beta }=\phi_{\beta ,j}\;D_{\beta m}^{T}b^{m},\;\;\;\;i=1,\;2,m=0,\ldots ,n-1.$$

Analogous representation is proposed for the pressures as follows:

(23) $$P_{\beta }\;=\;D_{\beta m}^{p}b^{m},\;m=0,\ldots ,n-1;\beta =1,\ldots ,N,$$

as well as for the fluid velocities. These interpolation (or approximation) functions inherent in the response function method (RFM) follow the series of the given flow problem solutions, where the input uncertainty parameter *b* is initially discretized in a uniform way within the given lower and upper bounds. One introduces several uniform subdivision of the interval [inf(*b*), sup(*b*)], getting the set {*b*_1_, *b*_2_,…, *b*_p_}, and iteratively solves the Navier–Stokes equations system for a discrete value of this parameter *b*. This parametrization is introduced in the following thermodynamic equilibrium equations with the additional upper index α = 1,…, *p*, whose natural value usually does not increase above one hundred.

As is well known, the basic idea behind the FVM is an application of the Gauss–Ostrogradsky divergence theorem to replace the volumetric integrals inherent in governing Equations (10)–(12), with the surface integrals rewritten for all the finite volumes, completely composing the entire computational domain. The contribution of each finite volume to the global equilibrium equation is represented by the contribution of its center, as well as its outer faces. It remarkably differs from the FEM and the boundary element method (BEM) discretization [37,39], in which the significance of each element traditionally depends upon the contributions of their nodal points.

**Figure 1:**
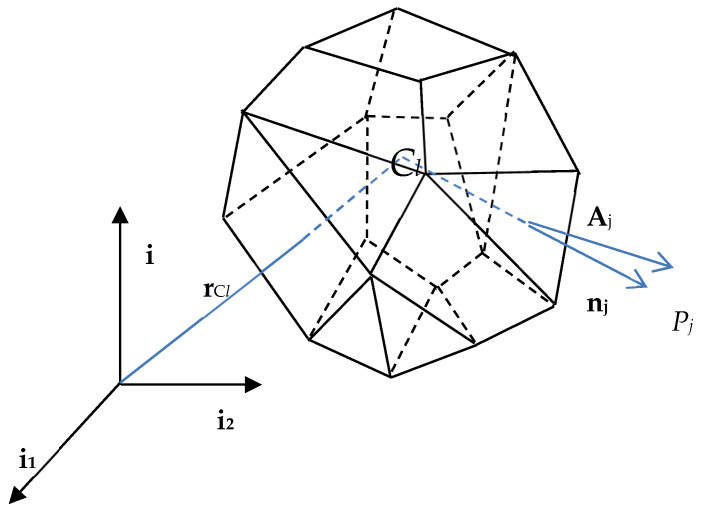
3D view of a single finite volume.

Therefore, Equation (10) is discretized in each local finite volume *l*, as follows [40,41,42]:

(24) $$\begin{array}{r} {\left(\frac{\rho^{\left(\alpha \right)}\Delta U^{\left(\alpha \right)}}{\Delta t}\right)}_{l}+\frac{1}{V_{l}}\sum_{j=1}^{n_{s}}\rho_{j}^{\left(\alpha \right)}U_{j}^{\left(\alpha \right)}U_{j}^{\left(\alpha \right)}A_{j}-\frac{1}{V_{l}}\sum_{j=1}^{n_{s}}\mu_{j}^{\left(\alpha \right)}\nabla U_{j}^{\left(\alpha \right)}A_{j}\\ ={\left(\nabla U^{\left(\alpha \right)}\right)}_{l}\nabla \mu_{l}^{\left(\alpha \right)}-{\left(\nabla P^{\left(\alpha \right)}\right)}_{l}+\rho_{l}^{\left(\alpha \right)}g^{\left(\alpha \right)} \end{array}$$

where *V_l_* denotes the *l*th finite volume (in Figure 1). The pressure gradient in x*_i_* direction is calculated with the use of the Gauss integration scheme, as follows [35,40]:

(25) $$\nabla P_{l}^{\left(\alpha \right)}\left(x_{i}\right)=\frac{1}{V_{l}}\sum_{j=1}^{n_{s}}P_{j}^{\left(\alpha \right)}A_{j}n_{j}$$

where *A_j_* is the area of the face *j*, *n_j_* denotes the versor of this surface directed outwards, and *α =* 1,*…*, *M*. An analogous procedure is proposed for the velocities, e.g.,

(26) $$\nabla U_{l}^{\left(\alpha \right)}=\frac{1}{V_{l}}\sum_{j=1}^{n_{s}}U_{j}^{\left(\alpha \right)}A_{j}n_{j}$$

where the central differencing scheme is applied to determine the given value at the cell face center. The following definitions are adopted, yielding:

(27) $$\left\{\begin{array}{c} K_{l}^{U\left(\alpha \right)}=\frac{\rho_{l}^{\left(\alpha \right)}}{\Delta t}+\frac{1}{V_{l}}\sum_{j=1}^{n_{s}}\left\{\left(1-\chi \right)\rho_{j}^{\left(\alpha \right)}U_{j}^{\left(\alpha \right)}A_{j}+\mu_{j}^{\left(\alpha \right)}\frac{A_{j}}{\left|d_{j}\right|}\right\}\\ {\bar{K}}_{lj}^{U\left(\alpha \right)}=\frac{1}{V_{l}}\left(\chi \rho_{j}^{\left(\alpha \right)}U_{j}^{\left(\alpha \right)}A_{j}-\mu_{j}^{\left(\alpha \right)}\frac{A_{j}}{\left|d_{j}\right|}\right)\\ Q_{l}^{U\left(\alpha \right)}=\frac{\rho_{l}^{\left(\alpha \right)}U_{l}^{\left(\alpha \right)}\left(t-\Delta t\right)}{\Delta t}-\frac{1}{V_{l}}\sum_{j=1}^{n_{s}}P_{j}^{\left(\alpha \right)}A_{j}n_{j}-\rho_{l}^{\left(\alpha \right)}g^{\left(\alpha \right)}\\ +\left(\nabla U_{l}^{\left(\alpha \right)}\left(t-\Delta t\right)\right)\left(\nabla \mu_{l}^{\left(\alpha \right)}\left(t-\Delta t\right)\right) \end{array}\right.$$

Finally, the following algebraic equations system is obtained for the *l*th finite volume:

(28) $$K_{l}^{U\left(\alpha \right)}U_{l}^{\left(\alpha \right)}\left(t\right)+\sum_{j=1}^{n_{s}}{\bar{K}}_{lj}^{U\left(\alpha \right)}{\bar{U}}_{lj}^{\left(\alpha \right)}\left(t\right)=Q_{l}^{U\left(\alpha \right)}$$

The variable ${\bar{U}}_{lj}^{\left(\alpha \right)}\left(t\right)$ is the so-called velocity face flux adjacent to the finite volume *l* and its *j* outer plane computed at time *t* for the response function test indexed with *α*. The global momentum equation in the RFM-based FVM is rewritten as follows:

(29) $$\sum_{l=1}^{N}K_{l}^{U\left(\alpha \right)}U_{l}^{\left(\alpha \right)}\left(t\right)+\sum_{l=1}^{N}\sum_{j=1}^{n_{s}}{\bar{K}}_{lj}^{U\left(\alpha \right)}{\bar{U}}_{lj}^{\left(\alpha \right)}\left(t\right)=\sum_{l=1}^{N}Q_{l}^{U\left(\alpha \right)}$$

The central differencing scheme, with the coefficient χ as the (linear) interpolation factor connecting the given finite volume and its particular face *j*, is introduced to evaluate the given scalar field at the cell face center. The continuity equation, cf. Equation (11), is discretized similarly on the finite volume level as follows:

(30) $$\sum_{j=1}^{n_{s}}U_{j}^{\left(\alpha \right)}A_{j}=0$$

Then, the following matrix equation for pressures (for the finite volumes center contribution and the finite volumes face, separately) is applied:

(31) $$K_{l}^{P\left(\alpha \right)}P_{l}^{\left(\alpha \right)}\left(t\right)+\sum_{j=1}^{n_{s}}{\bar{K}}_{lj}^{P\left(\alpha \right)}{\bar{P}}_{lj}^{\left(\alpha \right)}\left(t\right)=Q_{l}^{P\left(\alpha \right)}$$

where its global version is provided as follows:

(32) $$\sum_{l=1}^{N}K_{l}^{P\left(\alpha \right)}P_{l}^{\left(\alpha \right)}\left(t\right)+\sum_{l=1}^{N}\sum_{j=1}^{n_{s}}{\bar{K}}_{lj}^{P\left(\alpha \right)}{\bar{P}}_{lj}^{\left(\alpha \right)}\left(t\right)=\sum_{l=1}^{N}Q_{l}^{P\left(\alpha \right)}$$

Finally, the SFVM discretization of the heat transfer equation is proposed as follows:

(33) $$K_{l}^{T\left(\alpha \right)}T_{l}^{\left(\alpha \right)}\left(t\right)+\sum_{j=1}^{n_{s}}{\bar{K}}_{lj}^{T\left(\alpha \right)}{\bar{T}}_{lj}^{\left(\alpha \right)}\left(t\right)=Q_{l}^{T\left(\alpha \right)}$$

where

(34) $$\left\{\begin{array}{c} K_{l}^{T\left(\alpha \right)}=\frac{\rho_{l}^{\left(\alpha \right)}c_{l}^{\left(\alpha \right)}}{\Delta t}+U_{li}^{\left(\alpha \right)}\frac{1}{V_{l}}\rho_{l}^{\left(\alpha \right)}c_{l}^{\left(\alpha \right)}\sum_{j=1}^{n_{s}}\left\{\left(1-\chi \right)A_{lj}n_{lji}+k_{l}^{\left(\alpha \right)}\frac{A_{j}}{\left|d_{j}\right|}\right\}\\ {\bar{K}}_{lj}^{T\left(\alpha \right)}=U_{li}^{\left(\alpha \right)}\frac{1}{V_{l}}\rho_{l}^{\left(\alpha \right)}c_{l}^{\left(\alpha \right)}\chi A_{lj}n_{jli}\;,\;\;\;i=1,2,3\\ Q_{l}^{T\left(\alpha \right)}=\frac{\rho_{l}^{\left(\alpha \right)}c_{l}^{\left(\alpha \right)}}{\Delta t}T_{l}^{\left(\alpha \right)}\left(t-\Delta t\right)+\varphi_{l}^{\left(\alpha \right)} \end{array}\right.$$

and $\varphi_{l}^{\left(\alpha \right)}$ is the viscous dissipation in the *l*th finite volume and the *α*th RFM numerical test. Therefore, the global heat transfer equation for the SFVM yields [35] the following:

(35) $$\sum_{l=1}^{N}K_{l}^{T\left(\alpha \right)}T_{l}^{\left(\alpha \right)}\left(t\right)+\sum_{l=1}^{N}\sum_{j=1}^{n_{s}}{\bar{K}}_{lj}^{T\left(\alpha \right)}{\bar{T}}_{lj}^{\left(\alpha \right)}\left(t\right)=\sum_{l=1}^{N}Q_{l}^{T\left(\alpha \right)}$$

Having determined the series of the PVT solutions for the given flow problem, one needs to complete the polynomial representations for these solutions proposed in Equations (21)–(23), which can be achieved using an interpolation or approximation method. Usually, polynomial interpolation or the least squares method (LSM) is preferred, although some spline techniques or artificial intelligence tools can be engaged as well. Finally, the calculation of the first two probabilistic moments of the temperature begins analytically in the following way:

(36) $$E\left[T\left(b,x\right)\right]=\int_{-\infty }^{+\infty }T\left(b,x\right)\;p_{b}\left(t\right)dt=\int_{-\infty }^{+\infty }D_{\beta m}^{T}b^{m}\;p_{b}\left(t\right)dt$$

(37) $$Var\left(T\left(b,x\right)\right)=\int_{-\infty }^{+\infty }{\left(T\left(b,x\right)-E\left[T\left(b,x\right)\right]\right)}^{2}\;p_{b}\left(t\right)dt=\int_{-\infty }^{+\infty }{\left(D_{\beta m}^{T}b^{m}-E\left[T\left(b,x\right)\right]\right)}^{2}\;p_{b}\left(t\right)dt$$

In case of the existence of the abovementioned integrals, the results are identical in the probabilistic context, and such a methodology is called the semi-analytical method (SAM), due to the approximative character of the temperature representations. In general, one may apply the generalized Taylor expansion proposed by Equation (15), and then the expression describing expectations (Equation (17)) will be enriched with the approximating polynomials. This approach is usually called the stochastic perturbation technique, in which the tenth order is applicable in the given test flow problems. Finally, the statistical counterparts of Equations (36) and (37) are used in the classical Monte Carlo scheme, whose numerical accuracy is biased by the total number of random trials.

## 4. Numerical Simulation

### 4.1. The 2D Heat Transfer Benchmark Test

**Figure 2:**
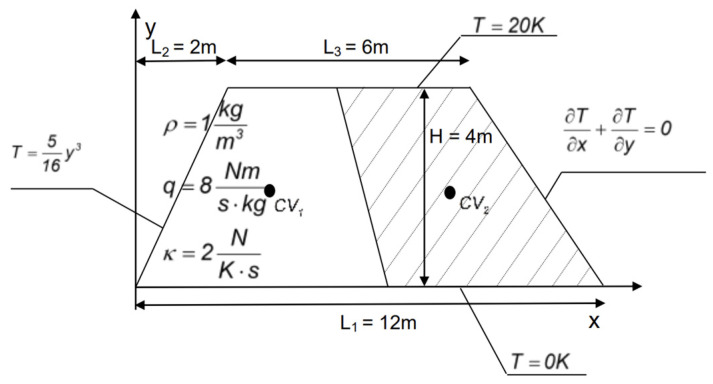
CVs configuration with boundary conditions in the 2D numerical test.

The first example shows the numerical solution of the heat transfer in a trapezoidal plate with two elementary finite volumes’ discretization. This benchmark example has been attached to validate computational implementation of the proposed SFVM and also to verify the numerical convergence of the entire procedure. This case study has been entirely coded in the MAPLE computer algebra system, where the FVM equations, Monte Carlo simulation relevant to the uncertainty quantification, as well as Shannon entropy computations have been compiled. The geometry of this benchmark test is shown schematically in Figure 2, where a constant source of heat $q=8.0\frac{N\;m}{s\;\mathrm{kg}}$ has been applied, and the material density of the plate equals $\rho =1.0\frac{\mathrm{kg}}{m^{3}}$, whereas the heat conductivity *κ* is the given Gaussian uncertainty source, and it has been identified using its first two probabilistic moments E[κ] = 2.0 N/K·s, while its coefficient of variation equals 0.05. The boundary conditions imposed on this plate are relevant to a heating of the upper surface and a maintenance of zero values at the lower surface, as shown in Figure 2.

An approximation of the integrals using the midpoint rule and the derivatives at the CV faces obtained using the second-order central differences yields the following algebraic system of equations:

(38) $$\left\{\begin{array}{c} 98T_{1}-17T_{2}=1386\;\\ 98T_{2}-17T_{1}=1746 \end{array}.\right.$$

The final deterministic solution for the two temperatures obtained in this case is obtained as follows:

(39) $$T^{0}=\left[\begin{array}{cc} \frac{2452}{69}\cdot \frac{1}{\kappa } & \frac{2884}{69}\cdot \frac{1}{\kappa } \end{array}\right].$$

Two polynomial functions relating the resulting temperatures with the heat conductivity *κ* have been determined using the polynomial interpolation function embedded in the *CurveFitting* library of the MAPLE system.

**Figure 3:**
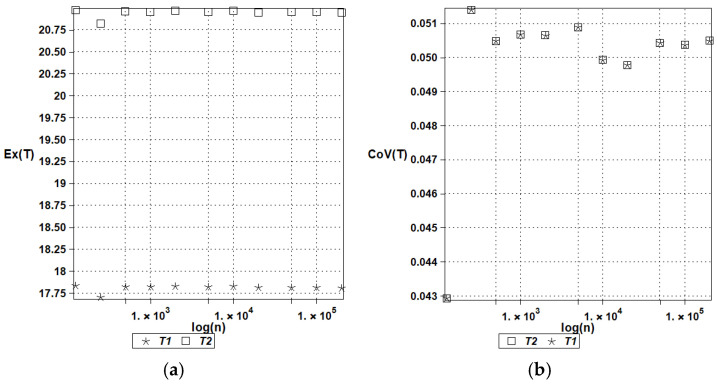
Expected values (**a**) and coefficients of variation (**b**) of the temperatures for Gaussian heat conductivity.

**Figure 4:**
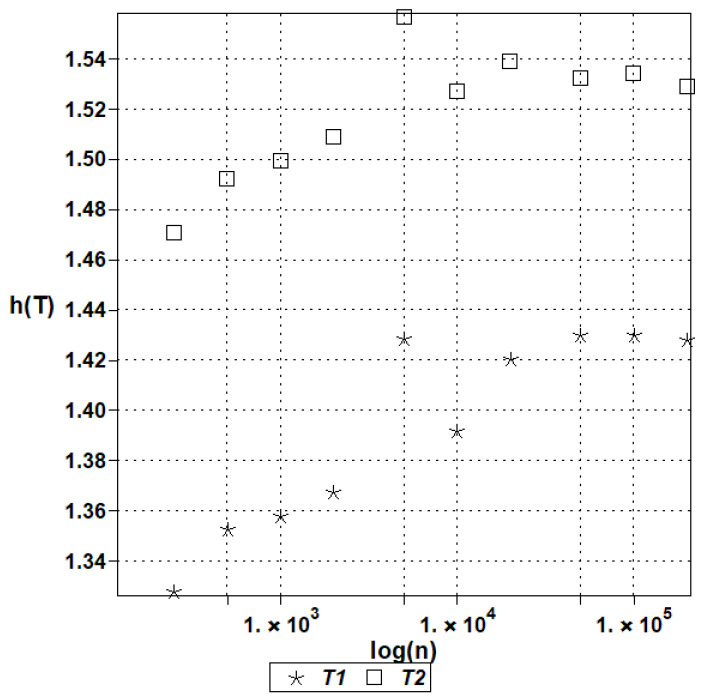
Shannon entropies of temperatures in the heat flow test for Gaussian heat conductivity.

Figure 3 presents the statistical estimators of the expectations (left graph) and the coefficient of variation (right graph) for the temperatures computed using the deterministic scheme of the finite volume method, in conjunction with the Monte Carlo simulation routines for 125, 250, 500, 1000, 2000, 5000, 10.000, 20.000, 50.000, 100.000, and finally, 200.000 random samples in the MAPLE system. The results of these simulations are shown in Figure 3, including the expectations of convergence (a) and the coefficients of variation convergence (b). Figure 4 illustrates the probabilistic convergence of both discrete temperatures; the horizontal axes in these graphs include a logarithm of an increasing MCS sample number. The first two moments have been estimated directly using the Statistics library of this system, while Shannon entropy has been determined by the partitioning of the histograms created in MAPLE; the additional script has been created for this purpose.

These two figures confirm that the fastest convergence is traditionally obtained for the lowest order probabilistic moment (expected value), where good accuracy is obtained even for about 10^3^ random trials. Satisfactory accuracy of the coefficients of variation is noticed while applying 5 × 10^4^ repetitions in the MCS approach. Reasonable convergence of Shannon entropy is determined with 2 × 10^5^, which means that reliable results in entropy analysis demand remarkably larger sizes of populations and computer effort. It is interesting that the coefficients of variation of both temperatures are almost the same, while the expectations and Shannon entropies display remarkably different values in the two discrete points under consideration. This partially confirms the previous observations made for nonlinear solids [43] that Shannon entropy shows the trends relevant to the expectations and coefficients of variation at the same time, so that instead of two graphs, a single uncertainty propagation graph may be analyzed.

**Figure 5:**
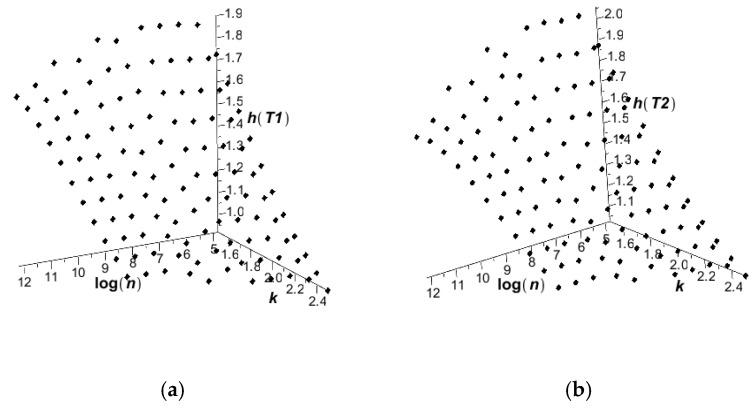
Shannon entropies of temperatures T_1_ (**a**) and T_2_ (**b**) in the heat flow test for Gaussian heat conductivity *k*, according to the random samples in the MCS analysis.

Additional observations for the Shannon entropies can be made while varying both the total number of random trials and the expectation of the randomized heat conductivity *k*, which is reported in Figure 5 below. This is separately delivered for the two given discrete volumes. This data confirms the high impact of the Shannon entropy to this expectation, whose increase remarkably decreases the entropy of interest. Furthermore, larger expectations of the heat conductivity results in a monotonous increase in the Shannon entropy, while increasing the size of the MCS analysis, and inversely, smaller expectations of random input cause a monotonous decrease in this entropy to its theoretical limit.

**Figure 6:**
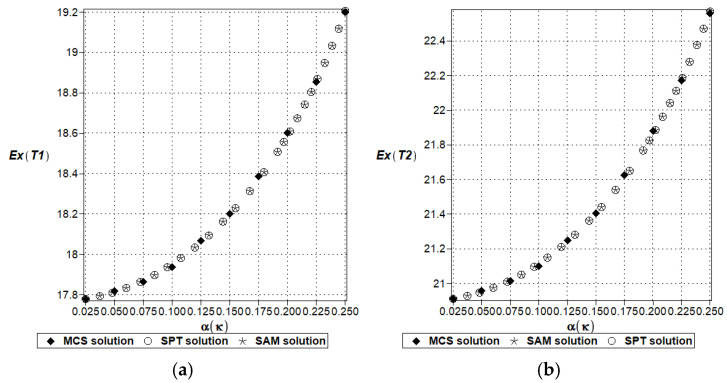
A comparison of the temperature expectations (T_1_ (**a**) & T_2_ (**b**)) in the given discrete volumes using three concurrent computational techniques.

**Figure 7:**
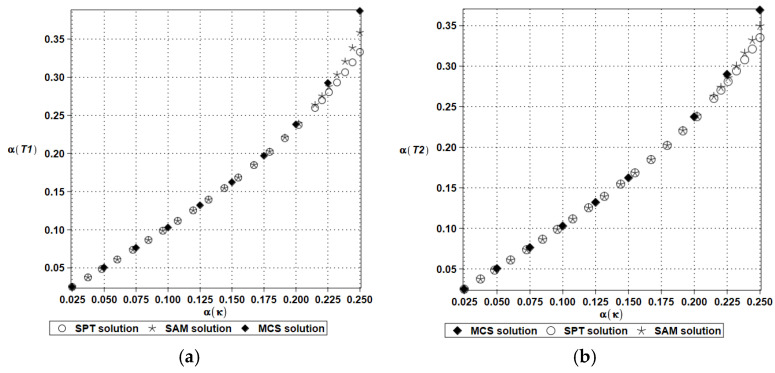
A comparison of the temperature coefficients of variation (T_1_ (**a**) & T_2_ (**b**)) in the given discrete volumes using three concurrent computational techniques.

This benchmark test has also been used to contrast the first two moment computations computed using the simulation method (MCS solution) with a semi-analytical approach (SAM solution), where symbolic integration has been delivered, and also with the stochastic perturbation approach of the tenth order (SPT solution). This study is attached to check a validity range of the SPT technique, whose time and computer power usage is close to those of the deterministic case study solution. The results are shown in Figure 6 and Figure 7 below for two discrete volumes in the function of an increasing input uncertainty in this model, i.e., α(κ) ∈ [0.00,0.25], which corresponds to a huge uncertainty regarding the temperature-independent physical characteristics of solids. Due to the nature of the specific solutions, the stochastic perturbation and semi-analytical methods return continuous functions with respect to this parameter α(κ) when the simulation for each discrete value α(κ) = 0.025*n, where natural *n* = 1, …, 10. The results cannot be deduced from Figure 6 and Figure 7.

The expected values contained in Figure 6a,b coincide perfectly with each other for all three methods and for any input uncertainty level. This coincidence is expected, even for lower-order stochastic perturbation theories; nevertheless, the existence of semi-analytical trend in these results depends upon specific stochastic responses of the given physical systems. Not all response functions (except polynomial types) can be relatively easily integrated using the Gaussian probability density function (PDF). This methodology may also fail when non-Gaussian PDF is chosen for a particular physical motivation; then, the application of polynomial approximations may not return the exact analytical result. These effects are also expected for higher-order statistics, for which symbolic integration is more complex. All the trends of both expectations are nonlinear, smooth, and convex, so one can also extrapolate from these curves a propagation of the expected values out of this interval. Figure 7 presents additional information concerning the second-order statistics (variance), where almost perfect agreement is restricted to the interval α(κ) = [0.00, …, 0.20].

Eventually, all the methods start to diverge from each other, and the largest values come from the MCS approach, with smaller values resulting from the analytical approach (SAM curve), while the SPT trend begins to underestimate this probabilistic moment. It is likely that the greatest precision in the SPT approach could be achieved by systematically increasing the order of this method while increasing the input uncertainty level above the critical value α(κ) = 0.20. One also notices that the general trend is almost linear from the very beginning, and then for about α(κ) = 0.20, it falls into a curvilinear mode, and the extrapolation for larger input uncertainties may be biased, with a higher modeling error; the second moments are remarkably close to each other for two given finite volumes. Concluding this benchmark test, one may assume that the usage of the time-consuming Monte Carlo simulation for numerical analysis of the basic probabilistic characteristics of the physical responses in linear systems with limited input uncertainty may be replaced with some alternative techniques, but this is not the case for Shannon entropy, where statistical estimation of the histogram remains the best approach.

**Figure 8:**
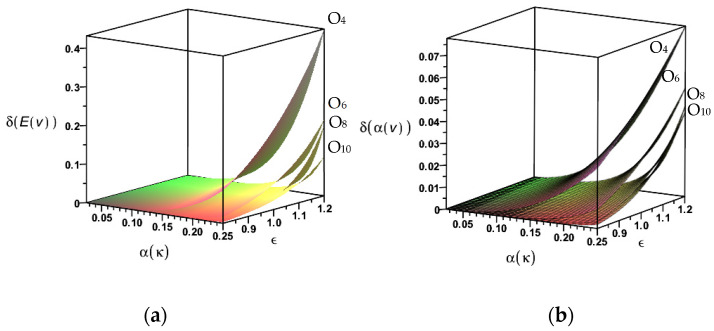
Common influence of the input uncertainty and the perturbation parameter on (**a**) expected values and (**b**) coefficients of variation of the temperature in the finite volume 1.

The final part of this numerical experiment has been devoted to the verification of the importance of the two input parameters inherent in the generalized stochastic perturbation technique—the input coefficient of variation of the heat conductivity and the perturbation parameters. The absolute differences between the fourth and the second *O*_4_, the sixth and the fourth *O*_6_, the eighth and the sixth *O*_8_, as well as the tenth and the eighth *O*_10_, are presented on Figure 8.

Both graphs well-document that the differences between the neighboring even orders of the Taylor expansions vanish rapidly when increasing the order of this methodology. These differences quite expectedly increase while increasing the input uncertainty level and the perturbation parameter value above the traditionally accepted value of one. The precision of the tenth-order perturbation scheme can be validated by contrasting these two figures with Figure 6a and Figure 7a, respectively. Taking into account ε = 1 and the extreme value of the input CoV equal to 0.25, it is observed that the modeling error in the expectations is remarkably smaller than 1%, which confirms the sufficient precision of the determination of the first two moments. An analogous error while computing the coefficient of variation for the same combination of input parameters equals about 2.5%; nevertheless, an application of the input CoV equal to 5% in the first case and 10% in the second case, makes this error negligible. This discussion may gain importance when considering temperature-dependent physical parameters, for which uncertainty propagation may be highly nonlinear during heating or coupled heat and mass transfer processes.

### 4.2. The 3D Coupled Lid-Driven Cavity Flow

**Figure 9:**
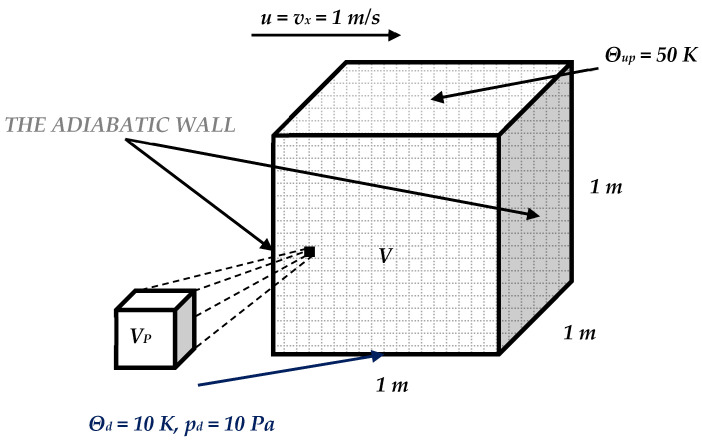
Boundary conditions for the lid-driven cavity flow.

Let us consider a cube of unit dimensions discretized into 400 equal cubic finite volumes containing a fluid with the following physical parameters—density, specific heat, thermal conductivity, and viscosity (both CoVs equal 0.10). These two parameters are randomized separately, according to the Gaussian distribution, to distinguish the influence of their uncertainty on the PVT solution of the given fluid flow problem. The boundary conditions introduced for this cube relevant to the forced uniform flow at the upper surface and the surface temperature difference between the upper and lower surface are schematically shown in Figure 9.

The problem is restricted to 2D analysis to provide a more apparent final visualization of the resulting state functions and their basic probabilistic characteristics. The time increment has been set as Δ*t* = 0.10 s, and the computations have been stopped after 10 s. It is clear that a composition of the physical parameters of the fluid is artificial and is taken to complete this benchmark test, while realistic fluids analysis would be more demanding. Computational analysis has been performed with hybrid usage of three different computer systems, namely (a) OpenFVM (a series of the few deterministic Navier–Stokes problem solutions, with some varying physical parameters) [44], (b) symbolic environment of the mathematical package MAPLE [45] (WLSM fitting), and (c) the freeware FEPlot 3.1 [35] (visualization of probabilistic moments and entropy).

**Figure 10:**
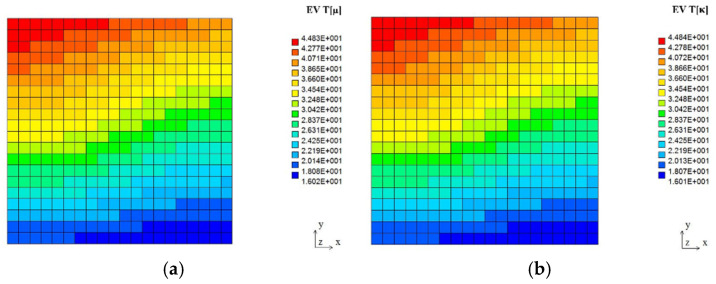
Expected values of the temperature field in the lid-driven cavity flow test for Gaussian viscosity (**a**) and heat conductivity (**b**).

**Figure 11:**
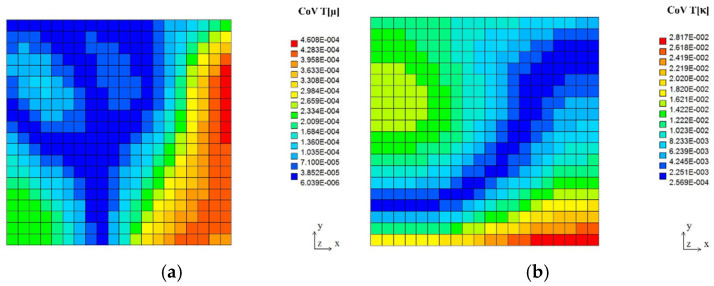
Coefficients of variation of the temperature field in the lid-driven cavity flow test for Gaussian viscosity (**a**) and heat conductivity (**b**).

**Figure 12:**
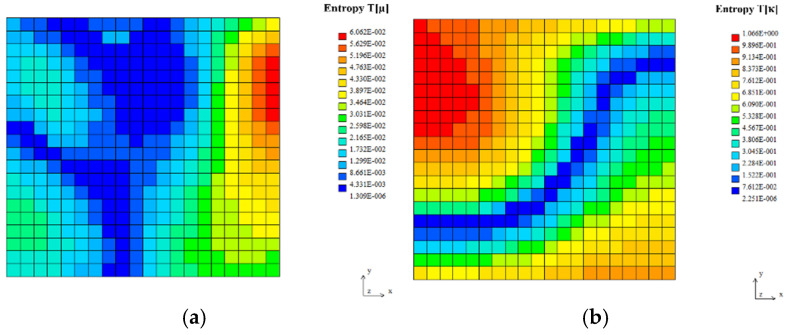
Shannon entropies for the temperature field in the lid-driven cavity flow test for Gaussian viscosity (**a**) and heat conductivity (**b**).

The expected values, coefficients of variation, and Shannon entropies of the resulting temperatures in two tests, including random viscosity (a) and heat conductivity (b), are shown in Figure 10, Figure 11 and Figure 12 below. It is rather natural and intuitively clear that the expected values of the temperatures in both problems are almost equal to each other (Figure 10), and they increase moderately from the upper to the lower surface of this quadratic cavity, resulting from the thermal boundary conditions presented in Figure 9. Negligible boundary temperature fluctuations throughout the upper and lower edges are caused by the rotational character of this particular flow.

First of all, it is noticeable that the coefficients of variation, as well as probabilistic entropies, exhibit similar spatial distributions; this similarity holds true for both uncertainty sources separately. These parameters are, at the same time, completely different from the corresponding distributions of the expectations.

Moreover, one notices that the uncertainty in fluid viscosity causes extreme uncertainty of the temperature distribution (the largest values of the CoV and entropy) in some part of the right vertical edge of this computational domain. Analogous uncertainty in the fluid’s heat conductivity results in the extremes near the upper left corner, quite close to the cavity inlet. Interestingly, the minimum values of the resulting temperature uncertainty (close to 0, which is adjacent to the deterministic situation) form almost the same patterns within the given 2D domain for the fluid viscosity and a very similar pattern to that of the random heat conductivity case. Finally, it is remarkable that larger CoVs and Shannon entropies are noticed while randomizing heat conductivity than the corresponding values obtained for fluid viscosity. It agrees well with engineering intuition and may serve as some verification of this probabilistic solution.

## 5. Concluding Remarks

(1) Shannon entropy determination for the PVT solution of the fluid flow problems with uncertainty, solved using the stochastic finite volume method, has been presented in this work. This approach uses fully coupled Navier–Stokes equations and dual probabilistic methodology, based on the generalized stochastic perturbation method, as well as the Monte Carlo simulation technique. It has been demonstrated here that the spatial distribution of probabilistic entropies is very close to the additional distribution of the coefficients of variation of the given fluid state function and may be useful in further uncertainty analysis for flow problems. This coincidence is observed for two different physical properties of the fluid, namely heat conductivity and viscosity, so the results do not appear to be accidental. Additionally, the probabilistic convergence of Shannon entropy has been documented using two discrete volumes, discretizing some planar heat conduction problems. Therefore, Shannon entropy would be advisable to illustrate uncertainty propagation in the flow problem instead of a series of the probabilistic moments and coefficients, which need to be studied together to achieve the same goal. It should be highlighted that contrary to the existing research in computational mechanics, this study enables the spatial distributions of Shannon entropy throughout the entire computational domain, and this entropy exhibits local characteristics connected with the discrete finite volume.
(2) The numerical solution presented here is based on the hybrid usage of the open source FVM program, a computer algebra system for probabilistic analyses, and the LSM fittings, as well as FEPlot software to complete a visualization of the resulting probabilistic moments and entropies. Further implementations focus on a closer interfacing of these three systems, as well as on the parameter sensitivity of the resulting entropy concerning the histogram partitioning, the Monte Carlo random trials number, the input uncertainty level, as well as the FVM time and the spatial discretization of the given flow problem. It may be that due to the numerical error of the solution itself or erroneous definition of the aforementioned problem parameter settings, Shannon entropy distribution computation would be inefficient. In case of any possible numerical discrepancies, other probabilistic entropy models could be considered.

## Data Availability

The original contributions presented in the study are included in the article, further inquiries can be directed to the corresponding author.
